# Summary of the Fibromyalgia Research Symposium 2016 in Nagasaki

**DOI:** 10.1097/PR9.0000000000000582

**Published:** 2017-01-31

**Authors:** Daniel J. Clauw, Hiroshi Ueda

**Affiliations:** a The University of Michigan, Ann Arbor MI, USA; b Department of Pharmacology and Therapeutic Innovation, Nagasaki University Graduate School of Biomedical Sciences, Nagasaki, Japan

The IASP Satellite Symposium on Fibromyalgia (FM) took place from October 1 to 2 in Nagasaki, Japan. The conference was co-hosted by Dr. Hiroshi Ueda of Nagaskai University and Dr. Daniel Clauw from the University of Michigan. The conference began with opening remarks from Dr. Ueda and then the Keynote address was delivered by Dr. Clauw. Dr. Clauw stated his opinion that the pathophysiology of FM is fairly well understood and is primarily due to central nervous system (CNS) dysfunction that leads to multifocal pain, as well as fatigue accompanied by sleep, memory, and mood difficulties. Dr. Clauw made the case that the CNS theory of FM is the only one that has moved past simply showing an association between findings and the presence of FM to showing that specific CNS abnormality (eg, abnormal levels of neurotransmitters in the CNS) targeted by drugs with specific mechanisms of action leading to improvement in the subset of individuals with FM who display that particular pathophysiological abnormality. The following talk was by Dr. Winfried Hauser, who emphasized the importance of psychosocial stressors in the development and worsening of individuals with FM. Dr. Kathleen Sluka presented both preclinical and clinical data, suggesting that FM was characterized by diffuse hyperalgesia/allodynia and that both the periphery and CNS may be contributing to symptoms such as fatigue. Dr. Jon Levine presented mainly preclinical studies, suggesting that both CNS and peripheral factors could potentially be playing a role in FM. Dr. Hiroshi Ueda then presented a series of preclinical studies using an intermittent cold stress model in mice that leads to the development of diffuse hyperalgesia and pain behaviors, reminiscent of those seen in FM, and responds to the same types of CNS-acting drugs that work in patients. Dr. Lars Arendt-Nielsen presented a series of studies performed by his group and others showing that the cardinal features of diffuse hyperalgesia/allodynia can be identified in subsets of virtually every chronic pain condition. Dr. Hiroshi Oka introduced the current status of FM treatment in Japan. The first day ended with 2 speakers in oral presentation. Dr. Chie Usui gave a talk about positron emission tomography study in FM, and Dr. Deepak Sharan gave a talk about the 5-year follow-up of individuals in a multidisciplinary FM treatment center in India.

The following day began with Dr. Claudia Sommer's presentation of her group's and others data showing that patients with FM have reduced intraepidermal nerve fiber density and postulated that this is playing a role in the pathogenesis of FM. Dr. Manuel Martinez-Lavin then presented his theory that FM is a sympathetically mediated pain syndrome. Dr. Roland Staud suggested that some of the CNS hyperalgesia in FM is reduced when individuals are infused with lidocaine, such that peripheral nociceptive input might be partly responsible for driving the central sensitization seen in FM. Dr. Eva Kosek then followed by presenting neuroimaging and quantitative sensory testing data, suggesting that the CNS hyperactivity in FM can be modulated by both pharmacological and nonpharmacological therapies. Dr. Richard Harris followed by showing evidence that the endogenous opioid system may be partly responsible for the CNS hyperactivity and symptoms seen in FM and suggested that this may explain why opioid therapy is often either ineffective in FM or sometimes actually leads to worsening of symptoms. The following oral presentation was by Dr. Kenji Miki who showed a phase IIa trial of mirtazapine which showed some evidence of efficacy in FM, and then Dr. Kenichi Osada presented a new device for detection of small fiber neuropathy. Abstracts of all presentations whose authors expressed an interest in participating in this report are presented below.

In summary, this internationally recognized set of speakers highlighted the fact that much is now known regarding the potential pathogenic mechanisms in FM. Nearly all agree that CNS factors play a predominant role in FM, but there are emerging data suggesting that peripheral factors may also play a role in some individuals and that these individuals might benefit from a different treatment plan than those with pure CNS disease.

## Overview of fibromyalgia clinical syndrome

### [Keynote Lecture]

Daniel J. Clauw

The University of Michigan, Ann Arbor MI, USA

The thinking regarding fibromyalgia (FM) has changed tremendously over the past 2 decades. Originally thought to be a discrete disorder, it is now clear that it might be better considered the end of a continuum, where the key clinical features are multifocal pain accompanied by somatic syndromes such as fatigue, sleep, and memory difficulties. Many but not all individuals with FM also have elevated levels of psychological distress—but both clinical and mechanistic studies clearly demonstrate that the key elements of fibromyalgia and psychological distress are independent of each other (but often co-occur). This independence of FM from psychological factors is especially true in individuals who have elevated high levels of “fibromyalgianess” (FMness—or centralized pain) but do not meet criteria for fibromyalgia. In these individuals with “sub-threshold FM,” there is a linear relationship between FMness and important clinical features (eg, nonresponsiveness to opioid analgesics and surgery), and this linear relationship only disappears when studies focus only on individuals at the end of this continuum (ie, those who meet criteria for FM).

The neurobiology of FM and centralized pain is becoming increasingly well understood, with abnormalities in levels of central nervous system (CNS) neurotransmitters, increases in pain and sensory responsiveness, and neuroimaging evidence of changes in functional connectivity as well as the size and shape of key brain structures. Nearly all of the treatments that are effective for subsets of patients with FM are directed toward these CNS abnormalities.

Fibromyalgia is now considered in the broader pain field to be the model for centralized pain, wherein pain and other symptoms are originating more so from the CNS than the periphery. Individuals with centralized pain are less responsive to classic and commonly used analgesic regimens (nonsteroidal anti-inflammatory drugs, opioids, injections, and surgery). They respond better to CNS-acting analgesics and aggressive use of nonpharmacological therapies aimed at the CNS. In summary, FM and centralized pain is “a different type of pain that requires an entirely different type of treatment.”

## [Lecture-1]

### The importance of psychosocial factors in the etiology and management of fibromyalgia

Winfried Häuser

Department of Psychosomatic Medicine and Psychotherapy, Technische Universität München, Germany

A model of interacting biological and psychosocial variables in the predisposition, triggering, and development of the chronicity of fibromyalgia syndrome (FMS) has been suggested. One prospective longitudinal population-based study demonstrated the association of lifestyle factors (sleep disturbances, obesity combined with low physical activity, and smoking) and the development of FMS. Workplace bullying and depression predicted the onset of FMS in one longitudinal cohort study each. A systematic review of case–control studies demonstrated an association of retrospective reports of childhood physical and sexual abuse and diagnosis of FMS in adulthood. Traumatic and major life events and long-term daily hassles might contribute—in genetically predisposed individuals—to central sensitivity and impaired descending pain inhibition.

The lifetime prevalence of mental disorders (anxiety, depression, and posttraumatic stress disorder) is up to 80% in patients with FMS. A systematic review of case series demonstrated that depression has a negative impact on FMS outcome (severity of symptoms and disability) and the success of multicomponent treatment. Two case series demonstrated that posttraumatic stress disorder has a negative impact on FMS outcome and working status.

A network meta-analysis demonstrated that drugs and cognitive behavioral therapies (CBTs) did not significantly differ in their efficacy at the end of treatment. However, the positive effects of drugs disappear after discontinuation within 2 weeks. In contrast, CBTs have a sustained reduction of pain, disability, and depression at 6-months follow-up (Cochrane review of randomized controlled trial). Finally, acceptance-based CBT was superior to the best available drug therapy (duloxetine and/or pregabalin) in improvement of quality of life at the end of treatment and at 6 months in a randomized controlled trial. Management of moderate and severe forms of FMS which are mainly due to mental comorbidities should include a mental health care specialist.

## [Lecture-2]

### Mechanisms underlying the transition from acute to chronic pain

Kathleen A. Sluka

The University of Iowa, Iowa, USA

The underling mechanisms involved in the transition from acute to chronic pain are critically important for preventing and treating chronic pain. Multiple stressors in a critical window may alter central neuron excitability and local immune responses. Neuronal plasticity in the nervous system and alterations in the local immune system by these stressors may underlie the development of chronic widespread pain. Dr. Sluka will present ongoing research examining the neurobiological mechanisms underlying the development of chronic widespread pain. She will show data examining sex differences and the protective role of testosterone, altered immune system activation in the muscle, and alterations in descending facilitation in animal models of chronic widespread pain.

## [Lecture-3]

### Fibromyalgia: the nerve of that disease

Jon D. Levine

Department of Medicine and Division of Neuroscience, University of California, San Francisco, USA

Fibromyalgia is a chronic widespread pain syndrome that currently has no pathognomonic feature or biomarker that allows diagnostic certainty and may present as a manifestation of another condition (secondary fibromyalgia). Thus, development of preclinical models provides a special opportunity to dissect underlying mechanisms. Given the importance of stress in fibromyalgia syndrome, we have focused on models of stress-induced pain. The results of these studies will be presented and the role of the neuroendocrine stress axis presented.

## [Lecture-4]

### Brain mechanisms and pharmacotherapy in generalized chronic pain model similar to fibromyalgia in mice

Hiroshi Ueda

Department of Pharmacology and Therapeutic Innovation, Nagasaki University Graduate School of Biomedical Sciences, Nagasaki, Japan

We have established a novel mouse model of generalized chronic pain model similar to fibromyalgia, using intermittent cold stress (ICS) exposure. This model was found to show long-lasting mechanical allodynia, thermal hyperalgesia, and other hypersensitivities to chemical and electrical stimuli. Female-predominant gender difference was observed only after the gonadectomy. In this talk, I will introduce a series of pharmacological studies and discuss underlying brain mechanisms and better pharmacotherapy. For the ICS exposure, mice were placed in a cold room at 4°C overnight (from 4:30 pm to 10:00 am), followed by ICS with alternating environmental temperatures between 24 and 4°C every 30 min from 10:00 am to 4:30 pm. These procedures were repeated twice. On day 3 (P1), the mice were returned to and adapted to a room temperature of 24°C for 1 hour before the nociception tests. In the ICS-induced generalized chronic pain model in mice, morphine had no significant analgesic effects when given in a systemic or intracerebroventricular (i.c.v.) route. Significant morphine analgesia was observed when NMDA receptor subunit, NR2A-deficient mice, or an NR2A antagonist (i.c.v.) was treated. The repeated co-administration of pregabalin with valspodar, an inhibitor of P-glycoprotein (p-gp), showed complete relief of pain at least for another week after the cessation of treatments. However, there was no morphine analgesia even after the complete recovery from chronic pain. The co-administration with the p-gp inhibitor also showed a potent potentiation of antihyperalgesia by duloxetine (intraperitoneal [i.p.]). In contrast, mirtazapine, another type of antidepressant (NaSSA) showed potent antihyperalgesic effects without the p-gp inhibitor, and repeated treatments showed complete relief from pain memory. After the repeated treatments with mirtazapine, morphine analgesic effects were recovered. Repeated treatments with donepezil also showed a complete relief from the chronic pain, but morphine analgesia was not recovered. I will discuss about brain mechanisms of all these pharmacotherapeutic effects.

## [Lecture-5]

### Mechanistic similarities between fibromyalgia and other chronic pain conditions

Lars Arendt-Nielsen

Center for Sensory-Motor Interaction, School of Medicine, Aalborg University, Aalborg, Denmark

Peripheral and central sensitization are important mechanisms for fibromyalgia (FM) and musculoskeletal pain conditions in general. Many similarities exist between different chronic musculoskeletal pain conditions. Musculoskeletal pain may transit from a localized pain problem through a regional representation to a widespread pain condition such as FM. As the pain condition transit from one to the other, more and more sensory abnormalities occur with many sensory abnormalities in FM. There is evidence that as well the intensity of ongoing pain as the duration of pain determine the degree of generalized hyperalgesia. This is important to realize as it underpins the importance of the ongoing nociception for the chronification process in conditions (eg, osteoarthrosis) where the peripheral nociceptive drivers are known, whereas it is more complicated in, eg, FM where the drivers are less obvious.

It is generally accepted that pain diagnosis and therapy should be mechanism based, and hence, pain assessment tools (pain biomarkers) should be sufficiently sensitive and advanced to provide such mechanistic information. Translating clinical observations to mechanisms and vice versa are not trivial, and tools to assess quantitatively the different phenomena are mandatory.

Such techniques for assessing the peripheral–central pain sensitization mechanisms in patients with FM and other patients with musculoskeletal pain have been developed and provide the opportunity to quantify pain mechanisms such as temporal summation, descending inhibition, spreading sensitization, and additional modality-specific hyperalgesic reactions. Such tools can help to phenotype patients with FM based on the role of the various pain sensitization mechanisms involved and have recently been used as tools to predict pain outcomes after pharmacological or surgical interventions in various groups of musculoskeletal pain conditions.

Relating clinical benefit of a given therapy with quantitative assessment of the pain sensitization mechanisms involved provides new opportunities for better diagnostics and hence for tailored and individualized management regimes.

Although assessed differently in specific tissues for various musculoskeletal pain conditions, the underlying mechanisms share common underlying features. This mechanistic understanding is of importance for developing better diagnostics and for implementing tailored pain management programs. The understanding that FM and other musculoskeletal conditions share common fundamental features has positioned FM as the one extreme end as opposed to, eg, a myofascial pain problem at the other end. This has provided some new insight into the development of the sensitization processes from one extreme to the other.

## [Lecture-6]

### Fibromyalgia treatment in Japan

Hiroshi Oka

Tokyo Rheumatism Pain Clinic, Tokyo, Japan

We have 3 double-blind multicenter placebo-controlled clinical trials for fibromyalgia (FM) in Japan. The first clinical trial was pregabalin (P-3). This trial demonstrated that pregabalin, at doses of up to 450 mg/d, was effective for the symptomatic relief of pain in Japanese patients with FM. Pregabalin also improved measures of sleep and functioning and was well tolerated. These data indicate that pregabalin is an effective treatment option for the relief of pain and sleep problems in Japanese patients with FM.

The second clinical trial was duloxetine (P-3). Duloxetine treatment was associated with improved outcomes in secondary and post-hoc analyses of the mean change in the BPI average pain score and most of the secondary outcomes, including analgesia and QOL. Duloxetine treatment was safe and well tolerated. These results suggest that duloxetine treatment could be associated with improvements in pain relief and QOL in Japanese patients with FM.

The third clinical trial was mirtazapine (P-2). Compared with placebo, mirtazapine produced a significantly greater analgesic effect and significantly greater improvements in QOL in terms of job ability, anxiety, and role or social functioning. Of note, mirtazapine was found to be effective in controlling FM pain even in patients without coexisting depression; other treatments are neurotropin, tramadol, and tramset in Japan.

## [Lecture-7]

### The role of peripheral nociceptors in fibromyalgia syndrome

Claudia Sommer

Department of Neurology, University of Würzburg, Germany

In recent years, data have accumulated showing pathoanatomical and functional abnormalities of peripheral nociceptors in patients with fibromyalgia syndrome (FMS). Taking together the cumulative data by several groups of researchers, this may be true for approximately 50% of the patients classified as the fibromyalgia group and may indicate an important subtype. In particular, quantitative sensory testing has shown deficits in thermal and tactile sensation in patients with FMS, indicating loss of function in A-delta and C-fibers or their afferent tracts. These data from the psychophysical testing are supported by findings from studies using evoked potentials like laser-evoked potentials and so-called pain-related evoked potentials, which show reduced amplitudes in patients with FMS. While these techniques do not allow a differentiation between a lesion in peripheral or central parts of the somatosensory system, skin biopsy and microneurography findings have clarified this issue. Confirmed by data from several laboratories, a subgroup of patients with FMS has a reduced intraepidermal nerve fiber density, indicating a peripheral pathology. This is supported by microneurography findings, where patients with FMS, like those with painful small fiber neuropathy, have increased spontaneous activity in nociceptors. Furthermore, patients with FMS have abnormal activity-induced slowing, which further differentiates them from patients with classical small fiber neuropathy. A possible explanation for this phenomenon is the finding that dermal C-fibers in patients with FMS are thinner than those of controls. A first potential explanation for the small fiber pathology of FMS has come from research on micro RNAs. We recently showed that miR-let-7d and its downstream target insulin-like growth factor-1 (IGF-1R) are aberrantly expressed in skin of patients with FMS with small nerve fiber impairment. Further research is needed to evaluate the significance of these findings for the pathophysiology of FMS.

## [Lecture-8]

### Fibromyalgia as a sympathetically maintained neuropathic pain syndrome

Manuel Martinez-Lavin

Department of Rheumatology, National Institute of Cardiology, Mexico

Our group has proposed that fibromyalgia is a neuropathic pain syndrome based on the following 3 arguments: (1) fibromyalgia is a stimulus-independent pain state; (2) the presence of allodynia as an essential feature of fibromyalgia; and (3) the presence of paresthesias as a distinctive feature of fibromyalgia. Among neuropathic syndromes, we propose that fibromyalgia pain is sympathetically maintained based on the following issues: the high frequency of physical, infectious, or psychological stressors as a triggering event; diverse heart rate variability studies showing that patients with fibromyalgia have changes consistent with ongoing sympathetic hyperactivity; a double-blind study demonstrating that norepinephrine injections rekindle fibromyalgia pain; the associated variations of the COMT gene and the adrenergic receptor gene, and finally, studies using the Composite Autonomic Symptom Score (COMPASS) questionnaire showing that fibromyalgia patients have prominent dysautonomia symptoms.

The recent finding of small fiber neuropathy in most patients with fibromyalgia reinforces the notion of fibromyalgia as a sympathetically maintained neuropathic pain syndrome. Corneal confocal microscopy has corroborated the presence of small nerve fiber pathology in fibromyalgia.

The SCN9A-encoded dorsal root ganglia sodium channels' (Na_v_1.7) genetic variations provide an additional link between fibromyalgia and small fiber neuropathy. Severe fibromyalgia is associated with the Na_v_1.7 rs6754031 GG genotype. However, gain-of-function mutations in sodium channel Na_v_1.7 are present in 28% of patients with small fiber neuropathy.

Outlook: Fibromyalgia is a sympathetically maintained neuropathic pain syndrome. Autonomic dysfunction provides a coherent explanation for the multiple nonpain-related fibromyalgia symptoms. Corneal confocal microscopy could become a useful noninvasive fibromyalgia diagnostic procedure. Dorsal root ganglia sodium channels are attractive therapeutic targets.

## [Lecture-9]

### Peripheral mechanisms of pain and fatigue in patients with fibromyalgia or chronic fatigue

Roland Staud

Department of Medicine, Rheumatology and Clinical Immunology, University of Florida, Gainesville, Florida, USA

Patients with musculoskeletal pain syndrome including fibromyalgia (FM) complain of chronic pain from deep tissues including muscles as well as often disabling fatigue. Previous research suggests the relevance of impulse input from deep tissues for clinical FM pain. Similarly, activated metaboreceptors of muscles seem to contribute to chronic fatigue.

We enrolled 62 female patients with FM and 58 patients with chronic fatigue into 2 double-blind controlled studies to receive 200 mg lidocaine or saline injections into the trapezius and gluteal muscles. In addition to pain and fatigue, study variables included pressure and heat hyperalgesia. Furthermore, all patients were asked to indicate by forced choice after the injections whether they had received the active study drug or normal saline.

Primary mechanical hyperalgesia at the shoulders and buttocks of FM and chronic fatigue syndrome subjects decreased significantly more after lidocaine than saline injections (*P* < 0.05). Similar results were obtained for secondary heat hyperalgesia at the arms (*P* < 0.05). After muscle injections, clinical FM pain and chronic fatigue significantly declined by 38% and 35%, respectively. According to postinjection questionnaires, subjects could not reliably distinguish whether they had received lidocaine or saline injections.

These results suggest that tissue injections can reliably reduce clinical FM pain and chronic fatigue and that peripheral impulse input is required for the maintenance of hyperalgesia and chronic fatigue in patients with chronic musculoskeletal pain.

**Acknowledgements:** These studies were supported by an NIH grant R01 NR014049-01 and NIH/NCATS Clinical and Translational Science grants UL1 TR000064. None of the authors have any financial or other relationships that might result in a conflict of interest.

## [Lecture-10]

### The effects of treatment on central pain modulation—lessons learned from functional magnetic resonance imaging studies

Eva Kosek

Department of Clinical Neuroscience and Osher Center, Karolinska Institutet and Stockholm Spine Center, Stockholm, Sweden

Fibromyalgia (FM) is associated with a generalized hypersensitivity to painful stimuli, and behaviour data indicate a dysfunction of descending pain inhibitory pathways. Therefore, central sensitization has been proposed to be an important pathophysiological mechanism in FM. The latter is supported by studies using functional magnetic resonance imaging demonstrating augmented nociceptive processing and reduced pain-related activation of rostral anterior cingulate cortex and thalamus, ie, cerebral regions implicated in pain modulation. These abnormalities were not associated with the degree of rated depression or anxiety, suggesting separate processing of negative affect and nociceptive input. In addition, patients with FM had less pain-related functional connectivity within the brain's pain inhibitory network and structural changes such as decreased cortical thickness and reduced brain volumes. There was an overlap between these functional and structural abnormalities, and both were more pronounced in patients with a longer duration of FM, suggesting a time-dependent progress of cerebral pathology in FM.

Furthermore, a short duration of FM predicted a better outcome after a 12-week treatment with the serotonin–noradrenalin reuptake inhibitor milnacipran. The degree of improvement and reductions in pain sensitivity after the milnacipran treatment corresponded to increased pain-related activation of precuneus, a cerebral area associated with pain modulation, and the default-mode network. In contrast, 12 weeks of cognitive behaviour therapy did not affect clinical pain or pain sensitivity but increased activations of cerebral regions implicated in executive cognitive control during painful stimulation and thus likely affected reappraisal of painful stimuli. Finally, a newly developed protocol of individually graded physical exercise for 15 weeks reduced symptoms and pain sensitivity. The elevated interstitial glutamate and pyruvate concentrations in the quadriceps muscle of patients with FM normalized after the exercise intervention. Pain-related cerebral activation was not affected, indicating that peripheral muscular mechanisms were involved in pain relief. However, a partial normalization of cerebral resting-state activity and improved cognitive functioning was seen in patients with FM after the exercise intervention. In conclusion, we demonstrated that at least some of the cerebral abnormalities in patients with FM are reversible. The fact that different treatment modalities affected specific cerebral mechanisms would support the rational for combination therapies in FM.

## [Lecture-11]

### Neural basis of multimodal sensory augmentation in fibromyalgia

Richard E. Harris

Department of Anesthesiology, University of Michigan, Ann Arbor MI, USA

Pain can be elicited throughout all mammalian sensory pathways, yet cross-modal sensory integration, and its relationship to pain in clinical conditions, has largely been unexplored. Fibromyalgia is the prototypical centralized chronic pain condition that is characterized by symptoms of multimodal sensory hypersensitivity, wherein individuals have aversive responses to touch/thermal stimuli, loud sounds, and even bright lights. As such, it is thought that this condition involves a large-scale amplification of sensory processes that cut across multiple modalities. Previous work suggests that the locus for this sensory integration may lie within the insular cortex as this structure has consistently displayed altered neurochemical imbalances as well as aberrant functional connectivity that have both been associated with the magnitude of clinical pain in this population. Here, I will present data demonstrating cross-modal sensory hypersensitivity to visual as well as pressure stimuli in patients with fibromyalgia and also investigate how changes in this activity can be modulated with pregabalin, a compound showing efficacy in this population. We will explore how functional magnetic resonance imaging has revealed that insular activity evoked by simple visual stimulation is intimately associated with chronic pain in fibromyalgia. Moreover, attenuation of this insular activity by the analgesic pregabalin is accompanied by concomitant reductions in clinical pain. These dynamic processes during pregabalin treatment occur in concert with reductions in insular glutamate (the brain's major excitatory neurotransmitter), gray matter volume, and functional connectivity between the default mode network (a constellation of brain regions activated during rest and self-referential thinking). Finally, I will also present data from multivariate classification methods using support vector machines applied to visual-evoked brain activity that can distinguish patients with fibromyalgia from healthy controls with significant accuracy. Interestingly, a separate support vector machine classification of treatment effects on visual-evoked activity can also identify patients administered with pregabalin as compared with placebo: importantly regions differentiating patients from controls and pregabalin from placebo treatment share overlapping insular topology. These data strongly suggest that abnormal integration of multisensory pathways within the insula may represent a pathophysiological mechanism in centralized chronic pain. Furthermore, insular response to aversive visual stimulation may also have utility as a marker for analgesic drug development beyond pregabalin, in particular for compounds that target the central nervous system.

This work is supported by grants from Pfizer Inc, the Dana Foundation, Department of Defense (Army Grant: DAMD-W81XWH-07-2-0050), and National Institutes of Health (NCCIH: P01 AT006663, R01-AT007550).

## [Oral-1]

### A study of brain metabolism in fibromyalgia by positron emission tomography

Chie Usui

Department of Psychiatry, Juntendo University Nerima Hospital, Japan

**Purpose:** The aim of this study was to determine the brain regions with altered metabolism in patients with treatment-naive fibromyalgia (FM).

**Methods:** We studied a total of 18 drug-naive patients with FM and 18 healthy controls without suffering from pain and who were matched for age and sex. [18F] fluoro-d-glucose positron emission tomography was tested in both patients with FM and controls. A voxel-by-voxel group analysis was performed using SPM8.

**Results:** No significant voxel (peak)-level result was detected in this study; however, some regions were detected as significant-size clusters. There was no significant difference in brain metabolism between patients with FM and controls. However, the right thalamus and left lentiform nucleus were hypermetabolic areas in patients with FM with poor prognosis compared with the healthy controls. In contrast, the left insula and left lentiform nucleus were hypometabolic areas in patients with FM with good prognosis compared with the healthy controls. Compared with patients with FM with good prognosis, FM patients with poor prognosis showed significant hypermetabolism in the left thalamus, bilateral lentiform nucleus, and right parahippocampal gyrus.

**Conclusion:** These findings suggest an association between the metabolism in the thalamus, lentiform nucleus, and parahippocampal gyrus and a prognosis in patients with FM. Further study with larger number of patients is required to confirm this finding.

This research is supported by the chronic pain research project from the Japan Agency for Medical Research and development, AMED.

## [Oral-2]

### Outcome of a sequenced multidisciplinary rehabilitation protocol for fibromyalgia—5-year follow-up

Deepak Sharan

RECOUP Neuromusculoskeletal Rehabilitation Centre, Bangalore, India

A study was conducted in 2009 to assess the outcome of a sequenced multidisciplinary rehabilitation for fibromyalgia syndrome (FMS) in 30 subjects (18 males and 12 females) at a follow-up of 1 year. A 5-year follow-up study was performed in the same individuals. All the subjects were diagnosed by an experienced orthopedic and rehabilitation physician and were treated with a sequenced multidisciplinary rehabilitation protocol. The outcomes measured included pain and sleep with visual analog scale, fatigue using Borg CR 10 scale, and depression with help of Beck Depression Inventory, Fibromyalgia Impact Questionnaire, and Short Form-36. Of the samples, 26 were software professionals, 3 were house wives, and 1 was a teacher. Nineteen subjects (12 males and 7 females) participated in the 5-year follow-up study. Eleven subjects had changed their contact details and could not be contacted. Mean scores during the first visit, at 1-year, and 5-year follow-ups were 6.56, 2.1, and 1.2 for pain, 5.36, 1.53, and 1 for fatigue, 5.7, 1.56, and 1 for sleeplessness, and 10.6, 4.7, and 2.64 for Beck Depression Inventory, respectively. Results of paired *t* test showed a significant difference (*P* > 0.05) between Fibromyalgia Impact Questionnaire and Short Form-36 scores of the first visit, 1-year, and 5-year follow-ups. Significant improvement (*P* > 0.05) in pain reduction, sleep, and fatigue was noticed after the 5-year follow-up. The subjects were not taking any specific treatment for FMS other than home-based exercises at the time of the last follow-up. This study emphasizes the need for a comprehensive sequenced multidisciplinary approach for effective treatment of FMS. There was a significant improvement in pain, sleep, fatigue, depression, and general quality of life, and the effects were maintained at a follow-up of 5 years.

## [Oral-3]

### Efficacy of mirtazapine for the treatment of fibromyalgia without concomitant depression: a randomized double-blind placebo-controlled phase IIa study in Japan

Kenji Miki^a^, Masato Murakami^b^, Hiroshi Oka^c^, Kaname Onozawa^d^, Sadahiro Yoshida^d^, Kenichi Osada^e^

^*a*^*Department of Pain Medicine, Osaka University Graduate School of Medicine, Center for pain management, Hayaishi Hospital*

^*b*^*Sanno Hospital, International University of Health and Welfare*

^*c*^*Department of Rheumatology, Hachioji Medical Center, Tokyo Medical University*

^*d*^*Clinical Research Planning Department, Meiji Seika Pharma Co, Ltd*

^*e*^*Department of Neuropsychiatry, St. Marianna University School of Medicine, Japan*

To evaluate the efficacy and safety of mirtazapine in Japanese patients with fibromyalgia (FM), a parallel-group randomized double-blind placebo-controlled phase IIa study was conducted at 57 sites. Patients aged 20 to 64 years who met the American College of Rheumatology 1990 diagnostic FM criteria and had stably high pain scores during a placebo run-in period were randomly assigned to receive mirtazapine (15 mg/d for 1 week and then 30 mg/d) or matching placebo for 12 weeks. The primary endpoint was change in mean numerical rating scale pain score from baseline to endpoint (week 12 or early discontinuation). Of 430 patients randomized (n = 215 each group), 422 (n = 211 each group) were analyzed for the primary endpoint. At the endpoint, mirtazapine caused a significantly greater reduction of mean numerical rating scale pain score compared with placebo (difference 0.44; 95% confidence interval, −0.72 to −0.17; *P* = 0.0018). The reduction by mirtazapine remained greater compared with placebo from week 6 onward. Mirtazapine also improved pain-related quality of life assessed by the Japanese version of the Fibromyalgia Impact Questionnaire and the Short Form-36 questionnaire. Adverse events were more common with mirtazapine than placebo (68.8% vs 56.7%), including somnolence (32.1% vs 7.4%), weight gain (17.7% vs 0.9%), and increased appetite (11.6% vs 3.3%). In conclusion, mirtazapine was an effective and safe treatment for Japanese patients with FM.

## [Oral-4]

### The investigation to the offset phenomenon for fibromyalgia to detect the pain with the medical instrument X

Kenichi Osada^a^, Yoshifuji Matsumoto^b^, Hirohiko Kuratune^c^, Syunpei Yokota^d^, Chie Usui^e^, Kenya NIshioka^f^, Masako Kikuchi^g^, Kusuki Nishioka^h^

^*a*^*Department of Neuropsychiatry, St. Marianna University School of Medicine*

^*b*^*Department of Rheumatology, Kuwana City Medical Center*

^*c*^*Department of Welfare & Health Science, Kansai Univ. Welfare Sciences*

^*d*^*Deartment of Pediatric Intractable Disease, Tokyo Medical Univ. Institute of Medical Science*

^*e*^*Department of Psychiatry, Juntendo Univ. Nerima Hospital*

^*f*^*Department of Neurology, Juntendo Univ. Hospital*

^*g*^*Department of Pediatrics, Yokohama City Univ. Medical Center*

^*h*^*Tokyo Medical University, Institute of Medical Science, Japan*

Offset analgesia responses were in a population of patients with neuropathic pain with small fiber neuropathy (Anesthesiology, M. Niesters, 2011;115:1063). We investigate the offset analgesia responses for the 119 patients with fibromyalgia by the pain detected with medical instrument X (detected small fiber neuropathy).This study was a prospective cohort study in multi-institutional joint research. Offset analgesia responses were measured by medical instrument X (Q-sense; Medoc).

Offset analgesia was diapered to patients with fibromyalgia, as the same as the patients with neuropathic pain. After offset, the pain of fibromyalgia was correlated with the total J-FIQ score (*P* < 0.001; *r* = 0.343). The peak value to the pain of the patients with depression (*n* = 5) was decreased compared with that of the controls (*n* = 17).

We considered that these data demonstrated that patients with fibromyalgia had the offset analgesia for the first time in the world. We considered that fibromyalgia might be included to the probable neuropathic pain syndrome.

## [Poster-1]

### Study on fibromyalgia patients with urinary complaints

Yuki Sekiguchi, Ayako Nakamura

Women's Clinic LUNA Group, LUNA Pelvic Floor Total Support Clinic, Department of Female Urology, Yokohama city, Japan

**Introduction:** Fibromyalgia is the disease that leads to various somatic complaints in addition to pain of the whole body. This time, patients with fibromyalgia with lower urinary tract complaints were studied.

**Patients and Methods:** Retrospectively, we reviewed the number of patients with fibromyalgia from January to March 2016 in the LUNA Pelvic Floor Total Support Clinic. Next, the patients with fibromyalgia were reviewed with or without urinary tract complaints. If patients had urinary tract complaints, they were checked for diagnosis by the urology department. In addition, the rates of efficacy of treatment were compared in the fibromyalgia group with and without urinary tract complaints. Regarding the diagnosis of fibromyalgia were used ACR1990, and the treatment efficiency was decided with the visual analog scale scale. We judged “effective treatment” if visual analog scale points decreased more than 2.

**Results:** There were 82 cases including first and revisits during 3 months in the LUNA Pelvic Floor Total Support Clinic. Twenty-nine (35%) patients had lower urinary tract complaints, of which there were 16 (65%) bladder pain syndrome or interstitial bladder cases, 8 (27%) nonneurogenic overactive bladder cases, 2 (7%) neurogenic overactive bladder cases.

The efficacy rate of treatment with and without lower urinary tract complaints was 83% and 62%, respectively. The patients with fibromyalgia with lower urinary tract complaints were relieved of pain easier than those without lower urinary tract complaints, but their lower urinary tract complaints got worse alternatively after pain relief.

**Conclusion:** Patients with fibromyalgia with lower urinary tract complaints could get pain control easier than those without lower urinary tract complaints, but their lower urinary tract complaints remain after pain relief.

## [Poster-2]

### Progressive brain changes in patients with chronic fatigue syndrome: confirmation of a longitudinal magnetic resonance imaging study by a larger cross-sectional study

Z. Y. Shan^a^, Richard Kwiatek^b^, R. Burnet^c^, P. Del Fante^d^, D. R. Staines^a^, S. M. Marshall-Gradisnik^a^, L. R. Barnden^a^

^*a*^*National Centre for Neuroimmunology and Emerging Diseases, Griffith University, Southport, QLD*

^*b*^*Modbury Hospital, Adelaide, SA*

^*c*^*Royal Adelaide Hospital, Adelaide, SA*

^*d*^*Healthfirst Network, Adelaide, SA, Australia*

Acceptance of fibromyalgia as a debilitating multidimensional medical disorder has been hampered by limited longitudinal research and lack of evidence of structural pathology. The chronic fatigue syndrome (CFS) is a disorder with considerable overlap of clinical features with fibromyalgia (Psychol Med 2002;32:881–8). The aim of this study was to uniquely assess for progressive regional and total brain volume changes in CFS. Twenty-five Canadian Consensus Criteria defined patients with CFS, and 25 matched healthy controls (HCs) had 3D gradient-echo brain magnetic resonance imaging scans performed (T1). Six years later, 15 CFS and 10 HCs of original study had repeat scans (T2). Twenty-five new CFS and 4 new HCs were recruited for an independent cross-sectional study (T3). Magnetic resonance imaging scans were interrogated using optimised (J Magn, Res Imaging 2016; DOI:10.1002/jmri.25283), whole-brain, SPM12 voxel-based morphometry (VBM). Cross-sectional between-group grey matter and white matter (WM) VBM analysis of T1, T2, and T3 revealed no significant differences. Voxel-based morphometry comparison of rates of change in WM volume between T2 and T1 for the 15 CFS and 10 HCs in T2 revealed a significant regional 4.5% reduction over 6 years in the posterior left inferior fronto-occipital fasciculus (corrected cluster *P*_FWE_ = 0.03, MNI co-ordinates −45, −20, −20). Cross-sectional between-group VBM of T2 plus T3 (40 CFS vs 14 HC) revealed significantly reduced regional WM volume in the left inferior fronto-occipital fasciculus in CFS (corrected cluster *P*_FWE_ = 0.005, MNI co-ordinates −52, −12, −16). For the first time, in any functional somatic syndrome, in any organ system, we have demonstrated progressive structural pathology, confirmed by a larger cross-sectional study. At the very least, these data indicate that patients with CFS experience a chronic organic brain syndrome. Notably, there exist no published reports of temporal lobe WM volume loss in depression, anxiety, or stress.

Supported by the Judith Jane Mason Foundation.

## [Poster-3]

### Nonclassical antidepressant mirtazapine shows long-lasting relief of generalized chronic pain and recovers morphine analgesia in an experimental fibromyalgia-like intermittent cold stress–model mice

Hiroshi Ueda, Takehiro Mukae, Hiroyuki Neyama, and Wakako Fujita

Department of Pharmacology and Therapeutic Innovation, Nagasaki University Graduate School of Biomedical Sciences, Japan

We have established a novel mouse model of generalized chronic pain model similar to fibromyalgia (FM), using intermittent cold stress (ICS) exposure. This model was found to show long-lasting mechanical allodynia, thermal hyperalgesia, and other hypersensitivities to chemical and electrical stimuli. Female-predominant gender difference was observed only after the gonadectomy. In a series of studies, we obtained the therapeutic effects of gabapentinoids and classical antidepressants, but not morphine in the ICS model. All these findings suggest the consistency to the clinical evidence for FM. In this study, we focus the beneficial effects of various types of antidepressants in terms of the site of action and therapeutic potentials. For the ICS exposure, mice were placed in a cold room at 4°C overnight (from 4:30 pm to 10:00 am), followed by ICS with alternating environmental temperatures between 24 and 4°C every 30 min from 10:00 am to 4:30 pm. These procedures were repeated twice. On day 3 (P1), the mice were returned to and adapted to a room temperature of 24°C for 1 hour before the nociception tests. Duloxetine and milnacipran showed potent antihyperalgesic effects when given intrathecally (i.t.) but not systemically (i.p.) or i.c.v. at P5. Mirtazapine, a nonclassical receptor antagonist-type antidepressant showed potent antihyperalgesic effects by i.p. injection. Its potent antihyperalgesic effects were also observed by i.c.v. injection but not i.t. injection. Repeated i.p. injections of mirtazapine from P5 to P11 caused the complete relief of abnormal pain even at P19, 7 days after the cessation of treatments. In addition, these treatments also recovered the morphine analgesic effects. All these findings suggest that repeated mirtazapine treatments cure the abnormal pain disease or pain memory in a FM-like animal model.

## [Poster-4]

### Beneficial effects by co-administration of pregabalin or duloxetine with P-glycoprotein inhibitor in intermittent cold stress–induced fibromyalgia-like model in mice

Takehiro Mukae, Hiroyuki Neyama, Yuki Morishita, Soichiro Kawamoto, Chiho Miyama, Ryoko Tsukahara, Hiroshi Ueda

Department of Pharmacology and Therapeutic Innovation, Nagasaki University Graduate School of Biomedical Sciences, Japan

We are looking for better pharmacotherapeutic treatments for generalized chronic pain disease by use of our established intermittent cold stress (ICS) model in mice. In a series of studies, we have demonstrated that the ICS model has many features similar to fibromyalgia in terms of pathophysiological (generalized chronic pain with female-predominant gender difference) and pharmacotherapeutic aspects (sensitive to gabapentinoids and antidepressants but not morphine). In the ICS exposure, mice were placed in a cold room at 4°C overnight (from 4:30 pm to 10:00 am), followed by ICS with alternating environmental temperatures between 24 and 4°C every 30 min from 10:00 am to 4:30 pm. These procedures were repeated twice. On day 3 (P1), the mice were returned to and adapted to a room temperature of 24°C for 1 hour before the nociception tests. In this study, we focused the beneficial potentials of P-glycoprotein (P-gp) inhibitors for the antihyperalgesic effects of pregabalin and duloxetine. Pregabalin (i.p.) showed potent antihyperalgesic effects in the ICS model. The combined treatment with valspodar, a P-gp inhibitor, decreased effective doses and prolonged the duration of pregabalin (i.p.)-induced antihyperalgesic effects. The repeated combined daily treatments of pregabalin and valspodar from P5 to P11 completely cured the basal hyperalgesic threshold 7 days after the cessation of drug treatments. Duloxetine showed potent antihyperalgesic effects when given i.t. but not i.p. The combination of duloxetine (i.p.) with tariquidal, another type of P-gp inhibitor, showed potent antihyperalgesic effects, and repeated treatments also cured the basal hyperalgesic threshold 7 days after the cessation of drug treatments. We are pursuing the mechanistic clarification of the P-gp–induced potentiation in terms of neurochemical and pharmacokinetic aspects.

## [Poster-5]

### Pathophysiological and pharmacotherapeutical actions in intermittent cold stress– and acidic saline–induced fibromyalgia/myalgia-like models in mice

Hiroyuki Neyama, Takehiro Mukae, Wakako Fujita, Hiroshi Ueda

Department of Pharmacology and Therapeutic Innovation, Nagasaki University Graduate School of Biomedical Sciences, Japan

We have established a novel mouse model of generalized chronic pain model similar to fibromyalgia (FM), using intermittent cold stress (ICS) exposure. This model was found to show long-lasting mechanical allodynia, thermal hyperalgesia, and other hypersensitivities to chemical and electrical stimuli. Female-predominant gender difference was observed only after the gonadectomy. In this presentation, we compared the pharmacological features in ICS model and intramuscular acidic saline model. Particularly in this presentation, we focus the therapeutic effects of donepezil and morphine. For the ICS exposure, mice were placed in a cold room at 4°C overnight (from 4:30 pm to 10:00 am), followed by ICS with alternating environmental temperatures between 24 and 4°C every 30 minute from 10:00 am to 4:30 pm. These procedures were repeated twice. On day 3 (P1), the mice were returned to and adapted to a room temperature of 24°C for 1 hour before the nociception tests (Nishiyori and Ueda, Mol Pain 2008;4:52). For the acidic saline model, it was as reported previously by Sluka et al. (Sluka et al. Muscle Nerve 2001;24:37–46). Intermittent cold stress model showed abnormal pain behaviors in thermal hyperalgesia, mechanical allodynia, and hypersensitivities to electrical, chemical (formalin i.p. or acetic acid i.p.), and muscle pain stimulation, respectively. Acidic saline model showed mechanical allodynia but not thermal hyperalgesia. To compare the pharmacological effects in both ICS and acidic saline models, mechanical paw withdrawal tests were performed at P5 when they were given i.p. or subcutaneously, respectively. Donepezil showed potent antiallodynic effects in the mechanical paw withdrawal tests in a dose range of 0.001 to 0.01 mg/kg (i.p.) at P5 in the ICS model. When donepezil was treated at P5, P7, and P9, the hyperalgesia was completely gone at P12 and P18. In the acidic saline model, however, donepezil shows much weaker effect with the median effective dose (ED_50_) ∼1 mg/kg (i.p.) at P5. On the contrary, morphine (s.c., i.c.v., and i.t.) did not show any antiallodynic effect in the ICS model, whereas it showed significant beneficial effects with 1 mg/kg s.c., 0.1 nmol i.c.v., and 1 nmol i.t., respectively in the acidic saline model. Pregabalin (i.p.) showed significant allodynic effects in both models. These results suggest that ICS model has more generalized pain symptoms and closer pharmacotherapeutic features to the clinical evidence in terms of lack of morphine analgesia than acidic saline model. To propose that donepezil is a new candidate for the treatment of FM, further basic and clinical information should be obtained.

